# Impact of *Acinetobacter baumannii* Superoxide Dismutase on Motility, Virulence, Oxidative Stress Resistance and Susceptibility to Antibiotics

**DOI:** 10.1371/journal.pone.0101033

**Published:** 2014-07-07

**Authors:** Magdalena Heindorf, Mahendar Kadari, Christine Heider, Evelyn Skiebe, Gottfried Wilharm

**Affiliations:** Robert Koch-Institute, Wernigerode Branch, Wernigerode, Germany; University of Illinois at Chicago College of Medicine, United States of America

## Abstract

*Acinetobacter baumannii* is a Gram-negative bacterium appearing as an opportunistic pathogen in hospital settings. Superoxide dismutase (SOD) contributes to virulence in several pathogenic bacteria by detoxifying reactive oxygen species released in the course of host defense reactions. However, the biological role of SODs in *A. baumannii* has not yet been elucidated. Here, we inactivated in *A. baumannii* ATCC 17978 gene A1S_2343, encoding a putative SOD of the Fe-Mn type by transposon insertion, resulting in mutant ATCC 17978 *sod2343::Km*. The mutation was also introduced in two naturally competent *A. baumannii* isolates by transformation with chromosomal DNA derived from mutant ATCC 17978 *sod2343::Km*. We demonstrate that inactivation of *sod2343* leads to significant motility defects in all three *A. baumannii* strains. The mutant strains were more susceptible to oxidative stress compared to their parental strains. Susceptibility to colistin and tetracycline was increased in all mutant strains while susceptibility of the mutants to gentamicin, levofloxacin and imipenem was strain-dependent. In the *Galleria mellonella* infection model the mutant strains were significantly attenuated. In conclusion, *sod2343* plays an important role in motility, resistance to oxidative stress, susceptibility to antibiotics and virulence in *A. baumannii*.

## Introduction

*Acinetobacter baumannii* is a Gram-negative, aerobic coccobacillus considered as an emerging nosocomial pathogen [1]–[3]. Particularly in intensive care units, *A. baumannii* is implicated in diverse infections including respiratory tract infections, bacteraemia, skin and soft tissue infections, urinary tract infections and meningitis. Its ability to withstand desiccation, disinfection and to form biofilms on abiotic surfaces, including medical devices such as catheters and ventilators, is believed to significantly contribute to survival and persistence of *A. baumannii* in the clinical environment [4]–[9]. Increasing rates of multidrug resistance in *A. baumannii* necessitate the development of new effective therapeutics alongside with efforts to reduce the selection pressure for resistance development [10]–[12]. Albeit virulence traits of *A. baumannii* are still poorly explored, significant progress has been made within the last years [4], [8], [13].

Superoxide dismutase (SOD) is an enzyme widely distributed in organisms and has been extensively studied in many species ranging from bacteria to human [14]–[17]. Superoxide dismutases (SODs) protect from the harmful effects of reactive oxygen species (ROS) by effectively catalyzing the conversion of superoxide radicals (•O_2_ ¯) into hydrogen peroxide and oxygen. SODs are metalloenzymes that are classified based on the metal cofactor used. One major class uses manganese or iron ions as catalytic metal (denoted as MnSODs and FeSODs). In bacteria, MnSODs and FeSODs are found in the cytoplasm and encoded by two distinct but related genes termed *sodA* and *sodB*, respectively [15], [18]. The second major class of SODs uses copper and zinc ions as catalytic metal (Cu-ZnSOD). Cu-ZnSODs encoded by *sodC* in bacteria are present in the periplasm of many Gram-negative bacteria, including *Escherichia coli* and *Salmonella* [16], [19]–[22]. Ni-containing SODs encoded by *sodN* were only lately discovered and seem to be less common [23].

As one facet of immune responses, pathogens to varying degrees are confronted with ROS in the course of infections. It is thus proposed that enzymes contributing to ROS detoxification including SODs can become important virulence factors [24], [25]. In a multitude of studies this conception was corroborated demonstrating attenuation of *sodA, sodB* and/or *sodC* mutants of various pathogens [15], [26], [27]. A contribution of ROS to control of infection with *A. baumannii* has been implicated [4], [28]. However, a contribution of ROS to macrophage-mediated killing of *A. baumannii* has recently been challenged [29].

A few years ago, an interrelationship between the bactericidal activity of antibiotics and the induction of ROS in bacteria has been proposed [30] and a *sodB* mutant of *E. coli* was described to exhibit increased susceptibility to norfloxacin [31]. Recently, it has been suggested that the bactericidal activity of polymyxins towards *A. baumannii* is mediated by hydroxyl radical production [32]. Very recently, however, the conception of ROS contributing to bactericidal activity of antibiotics was challenged in that no correlation between ROS production in bacteria and antibiotic treatment was found [33], [34].

Whole-genome sequencing of *Acinetobacter baumannii* ATCC 17978 revealed the presence of two *sod* genes designated A1S_2343 and A1S_3143 encoding putative Fe-Mn and Cu-ZnSOD, respectively [35]. Very recently, Mendez et al. [36] demonstrated that both copies are expressed in a multidrug-resistant strain and the proteins detectable in the culture supernatant. To date, the roles of *sod* genes of *A. baumannii* in motility, antibiotic resistance and virulence have not been elucidated. Here, we demonstrate for the first time a role of *A. baumannii* A1S_2343, a putative *sodB* gene [37], in motility, pathogenicity and resistance to oxidative stress and antibiotics.

## Materials and Methods

### Bacterial strains and culture conditions

*A. baumannii* strain ATCC 17978 was purchased from LGC Promochem and naturally competent clinical isolates were described recently [38], [39]. All strains were grown in Luria-Bertani (LB) broth or on LB agar at 37°C. Kanamycin at 10 µg/ml was added to media to maintain the *sod2343::Km* mutant derivatives if not otherwise stated. Ampicillin at 100 µg/ml was added to maintain complementation plasmid pWH*sod2343* and backbone plasmid pWH1266, respectively, if not otherwise stated.

Growth curves were determined as follows. Overnight cultures were grown at 37°C in LB medium supplemented with antibiotics as above. The overnight cultures were adjusted to 1 OD (600 nm) in LB medium without antibiotics and the OD-adjusted cultures used as inoculum for further cultivation in either disposable 10 ml-tubes or 250 ml-baffled flasks. In 10 ml-tubes, 2 ml of LB medium was inoculated with 50 µl of OD-adjusted inoculum, in 250 ml-baffled flasks 50 ml of LB medium was inoculated with 1 ml of OD-adjusted inoculum. All cultures were subsequently incubated at 37°C under constant shaking at 150 rpm for 8 hours. The OD at 600 nm was determined every hour. To this end, cultures grown in disposable culture tubes were directly measured in a Beckman Coulter DU720 spectrophotometer. From flask-based cultures 1 ml was sampled for OD measurement. For each strain, data obtained from three independent cultures grown on the same day were averaged. Statistical significance was tested by the Student's *t* test (two-tailed, unpaired) for the last three time points separately and *p* values below 0.05 were considered significant.

### Transposon mutagenesis, mutant screening and identification

Transposon mutagenesis of *A. baumannii* ATCC 17978, screening for motility-deficient mutants and identification of transposon insertion sites was performed as described [38]. In brief, preformed transposon/transposase complexes of EZ-Tn5 <KAN-2> (Epicentre Biotechnologies, USA) were transformed into electro-competent ATCC 17978. Transformants were selected on LB agar plates supplemented with 6 µg/ml kanamycin and subsequently screened for motility phenotypes on 0.5% agarose plates as described [38]. Following established protocols, single-primer PCR and subsequent DNA sequencing was conducted to identify the transposon insertion site of selected mutants. In mutant 52, insertion of the transposon was localized after nucleotide position 454 within the open reading frame A1S_2343 *(sod2343::Km*). The insertion was confirmed using forward primer 5′-CCATATGGCTATGATGATTTAGCG-3′ and reverse primer 5′-TGCAAGTTTTGCATTTGCATAGTC-3′, yielding a PCR product of 573 bps from wild type template and a product of 1794 bps from the *sod2343::Km* mutant template (see Fig. S1).

### Inactivation of *sod* in naturally competent *A. baumannii*

To construct *sod* mutants of naturally competent isolates 07–095 and 07–102, chromosomal DNA of the *sod2343::Km* mutant derivative of ATCC 17978 (mutant 52) was used for transformation. Chromosomal DNA of ATCC17978 *sod2343::Km* was purified using the MasterPure DNA purification kit (Epicentre Biotechnologies). A single colony of the respective isolate to transform was resuspended in 20 µl of sterile PBS and the suspension mixed in a ratio of 1∶1 (v/v) with the chromosomal DNA of ATCC17978 *sod2343::Km* (200 ng/µl). The mixture was stab-inoculated (7 times with 2 µl each) into a 0.5% agarose plate as described [39]. Then the plates were incubated overnight at 37°C. The next day, the bacteria were flushed off with 1 ml of sterile PBS and 100 µl of the suspension was spread on 1% agar plates containing 10 µg/ml of kanamycin for selection. Single colonies were picked from the kanamycin selective plates and transferred for regrowth on a secondary plate before subjecting to PCR for confirmation of gene inactivation (*sod2343::Km*) using forward primer 5′-CCATATGGCTATGATGATTTAGCG-3′ and reverse primer 5′-TGCAAGTTTTGCATTTGCATAGTC-3′.

### Construction of complementation plasmid pWH*sod2343*

Using forward primer 5′-ATTAGGATCCTCTTTTTTTCAATCTGTGTTATGCG-3′ and reverse primer 5′-ATTAGGATCCAATCTGATGCGCATTTTATGGATG-3′ we amplified by PCR a genomic region of *A. baumannii* ATCC 17978 encompassing nucleotides 2714817 to 2715729. This region includes A1S_2343 as well as the putative promotor and terminator region of the gene as identified using the softberry package available online (http://linux1.softberry.com/berry.phtml). The PCR product was digested with BamHI (cleavage sites introduced via primers as indicated by underline) and ligated into BamHI-digested vector pWH1266 [40]. The resulting complementation plasmid was termed pWH*sod2343*. Transformation and selection of the plasmid followed established protocols [38].

### Motility assays

To investigate the impact of *sod2343::Km* inactivation on motility, motility assays were performed for all *sod2343::Km* mutants and their respective parental strains. Motility was analysed on semi-solid agarose (0.5%) supplemented with 5 g/l tryptone and 2.5 mM NaCl in polystyrene Petri dishes [38]. Single colonies were spotted on agarose plates by puncturing the agarose layer. Then plates were sealed with parafilm to prevent drying and incubated overnight at 37°C. If not otherwise stated, the plates were analysed and documented after 18 hours of incubation. At least three independent replicates were performed.

### Infection in *Galleria mellonella* caterpillars

*Galleria mellonella* infections were performed as described [38]. *G. mellonella* caterpillars were purchased from Reptilienkosmos.de, Niederkrüchten, Germany. Bacterial strains were cultured overnight in 5 ml LB media at 37°C. Overnight cultures were diluted 1∶50 in LB and cultured for another 3 hours at 37°C. Bacteria were then washed and resuspended in sterile phosphate-buffered saline (PBS). Optical density (OD_600 nm_) was adjusted to 0.2 and then 5 µl of the bacterial suspension corresponding to 3×10^5^ CFU was injected into each *G. mellonella* larva through the last left proleg. For each infection experiment, groups of 16 caterpillars were assigned to each of the mutant and parental strains and two control groups were used, one treated with PBS and one untreated. Then infected caterpillars were incubated at 37°C and vitality of infected caterpillars was checked every day for a period of 5 days by touching the larvae and survival count was recorded. Caterpillars were considered dead when they showed no response to touching. Results were not considered valid when more than two dead caterpillars were found in control groups within five days. Three independent infection experiments were conducted.

Recovery of bacteria from the hemocoel of *Galleria* larvae immediately after injection (“immediate recovery assay”) was performed as follows. Groups of 10 larvae were treated as above with 5 µl of bacterial suspensions corresponding to 1.5×10^6^ CFU. Directly after injection of the 10 larvae within a group (taking 10 minutes) the larvae were frozen at −80°C for at least 1 hour. Frozen individual larvae were put into 1.5 ml tubes and homogenized in 300 µl of sterile PBS using a sterile glass rod. An aliquot of 100 µl was then subjected to a dilution series (10^−1^, 10^−2^, 10^−3^) that was plated on CHROMagar Acinetobacter (CHROMagar, France), the latter effectively suppressing growth of the *Galleria* microbiome. After incubation for 24 h at 37°C the CFU numbers were determined. Growth of *sod2343::Km* mutants on CHROMagar Acinetobacter was controlled to be unbiased in comparison to parental strains. Statistical significance was tested by the Student's *t* test (two-tailed, unpaired).

### Determination of sensitivity to reactive oxygen species (ROS)

Sensitivity of *sod2343::Km* mutants and their respective parental strains to H_2_O_2_ and paraquat was determined by the disc diffusion method. Agar plates (nutrient broth) were covered completely with appropriate cultures (OD_600 nm_ of approximately 0.5) and the liquid was removed. Then sterile paper discs were placed on the plates and loaded with 5 µl of H_2_O_2_ solutions (0%, 1%, 5% and 10%) and paraquat (5 µl of 5 mg/ml), respectively. Then plates were incubated overnight at 37°C. After 18 hours in the incubator, the sensitivity of mutants and their parental strains to H_2_O_2_ and paraquat was determined by measurement of the inhibition zone. At least three independent experiments were performed. Statistical significance was tested by the Student's *t* test (two-tailed, unpaired) and *p* values below 0.05 were considered significant.

### Determination of susceptibility to antibiotics

Agar plates (Luria-Bertani broth) were flushed with appropriate cultures as described for the disc diffusion method above and Etest strips (Liofilchem, Italy) were deposited for determination of minimal inhibitory concentrations (MIC) after incubation for 18 hours at 37°C. At least three independent MIC determination experiments were performed. Statistical significance was tested by the Student's *t* test (two-tailed, unpaired) and *p* values below 0.05 were considered significant.

### Recombinant production of a GST-SOD2343 fusion protein and generation of a polyclonal antiserum

The coding sequence of A1S_2343 of *A. baumannii* ATCC 17978 was amplified by PCR with forward primer 5′-AATTGGATCCATGACAACCATTACTTTACCTGCAC-3′ introducing a *BamH*I site (underlined) and reverse primer 5′-AATTGCGGCCGCTTATTTCTCTACACCAGCTGGTTGGC-3′ introducing a *Not*I site (underlined). The PCR product was cleaved with *BamH*I and *Not*I and introduced into expression vector pGEX-6P3 (GE Helathcare). Expression of the resultant GST-SOD2343 fusion protein was induced with 1 mM IPTG in expression host *E. coli* BL21 and purified on glutathione-sepharose 4B purification modules according to the manufacturer' recommendations (GST SpinTrap; GE Helathcare). The purified GST-SOD2343 fusion protein was dialyzed against PBS and used for immunization of a rabbit (Pineda Antikörper-Service, Berlin, Germany). After primary intradermal immunization with 150 µg of GST-SOD2343 using Freund's complete adjuvant, the rabbit received three boost injections subcutaneously at day 20, 30 and 40 with 150 µg of GST-SOD2343 each, using Freund's incomplete adjuvant. After bleeding on day 60, the serum obtained after clotting and centrifugation was collected, stored at −80°C and used at a dilution of 1∶5000 for immunoblotting.

### Superoxide dismutase activity assay

SOD activity was determined using the RANSOD assay (Randox Laboratories, UK) following the manufacturer's recommendations. The method is based on the generation of superoxide radicals using the xanthine oxidase (XOD) and xanthine as its substrate. The superoxide radicals react with 2-(4-iodophenyl)-3-(4-nitrophenol)-5-phenyltetrazolium chloride to produce a formazan dye which can be detected spectrophotometrically at 505 nm. In the presence of SOD activity, the dye formation is inhibited due to the consumption of superoxide radicals, and one unit of SOD is defined as a 50% inhibition of dye formation. Appropriate cultures grown in LB medium at 37°C overnight were adjusted to 1 OD (600 nm) the next day. 100 ml of the OD-adjusted suspensions were centrifuged (10 min, 5.000 g) and bacteria resuspended in 10 ml of sterile PBS. The cells were then disrupted using an EmulsiFlex-C3 (Avestin) homogenizer. After centrifugation (30 min, 20.000 g) the cleared supernatant was used for activity assays. To this end, 10 µl of cleared supernatant was mixed with 340 µl of xanthine substrate provided by the manufacturer, the reaction started by addition of 50 µl of XOD solution and recorded at 505 nm in a Specord 50 photometer (Analytic Jena) for 3 min. Calibration series were conducted at the day of sample measurement according to the manufacturer's recommendations.

## Results

### Inactivation of A1S_2343 in *A. baumannii* ATCC 17978 and subsequent transformation of naturally competent isolates of *A. baumannii*

In an attempt to identify genes involved in surface-associated motility of *A. baumannii* we performed transposon mutagenesis of *A. baumannii* ATCC 17978 and screened for motility-deficient mutants as recently described [38]. In the process, we identified a mutant with a transposon inserted into A1S_2343 encoding a putative Fe/Mn superoxide dismutase [35], later designated Fe SOD [36] and the gene correspondingly termed *sodB* [37]. In order to characterize the role of this SOD in *A. baumannii*, the homologues of A1S_2343 were inactivated in naturally competent isolates 07–095 and 07–102 [39] by allelic exchange with *sod2343::Km* from ATCC 17978. The inactivation was verified by PCR (Fig. S1).

**Figure 1:**
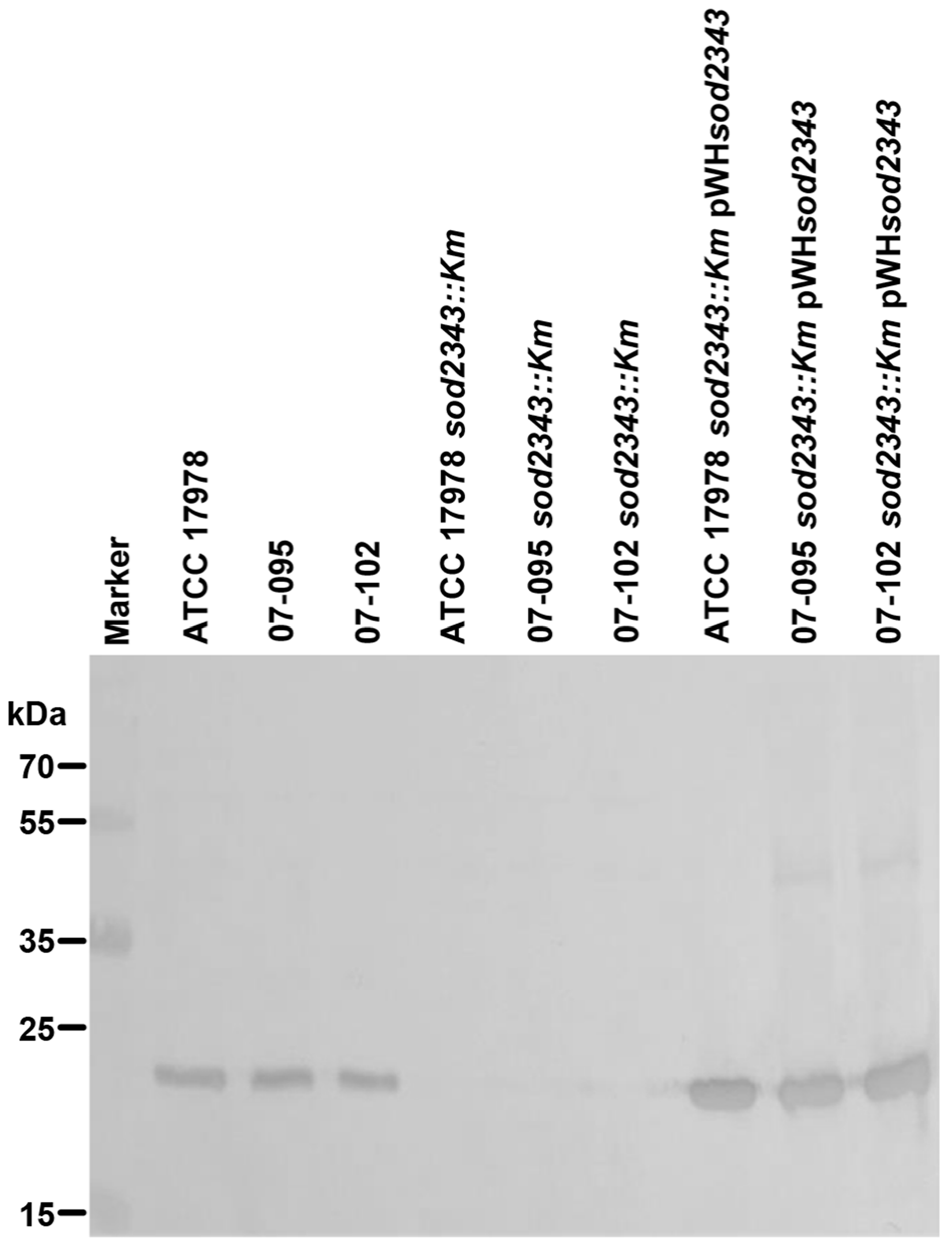
Confirmation of *sod2343::Km* mutants by immunoblotting. Overnight cultures as indicated were diluted 1∶20, grown for another 4 hours at 37°C, then adjusted to 1 OD (600 nm) and 0.5 ml of each was centrifuged and the pellet resuspended in 50 µl of loading buffer. 10 µl of each sample was loaded on an SDS-PAGE that was subsequently electro-blotted. A polyclonal antiserum raised against GST-SOD2343 fusion protein was diluted 1∶5000 for detection. The blots shown are representative of three independent replicates.

**Figure 2:**
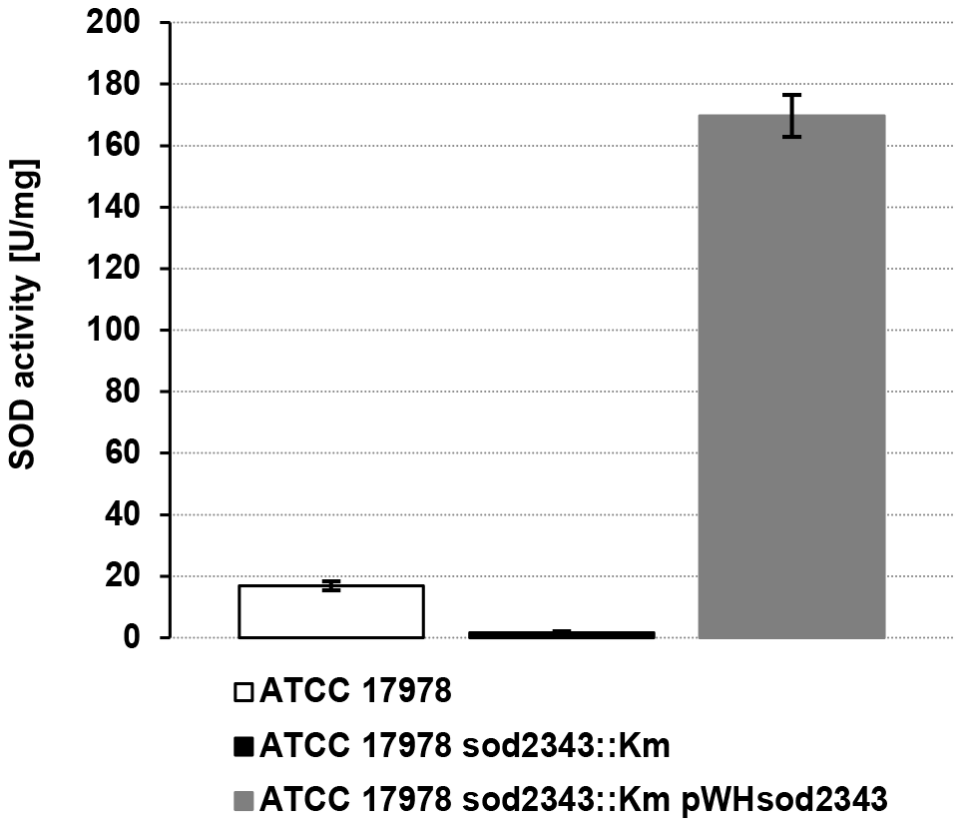
SOD activity assay reveals a minor residual activity of the ATCC 17978 *sod2343::Km* mutant and an overproduction of SOD2343 in the complemented strain compared to the parental one. OD-adjusted suspensions of indicated strains cultivated overnight were disrupted and after centrifugation cleared lysate supernatants were directly used for SOD activity assays as outlined in the Materials & Methods section. For each strain, data obtained from three independent cultures were averaged; error bars represent plus/minus one standard deviation.

To further confirm the inactivation of *sod2343* on the protein level, a polyclonal antiserum was raised against recombinantly produced SOD2343 protein and used for immunoblotting (Fig. 1). A protein with an approximate molecular mass of 22 kDa was detected in the parental strains but not in the *sod2343::Km* mutant derivatives (Fig. 1). This is well in accordance with the calculated molecular mass of the protein encoded by A1S_2343 which is 22.9 kDa, thus confirming detection of SOD2343 in the parental strains and proper inactivation of *sod2343* in the mutants. In addition, a plasmid harbouring A1S_2343 (pWH*sod2343*) was constructed for trans-complementation of the mutants. Immunoblotting (Fig. 1) demonstrated the successful trans-complementation of the mutants giving rise to an immuno-reactive protein of the same size as in the parental strains. However, the amount of SOD2343 protein detected in the complemented mutants was considerably above the level found in the parental strains. This was probably due to a copy-number effect of the complementation plasmid. To further substantiate this finding we performed SOD activity assays with cellular lysates obtained from ATCC 17978, its *sod2343::Km* derivative and the complemented mutant ATCC 17978 *sod2343::Km* pWH*sod2343* (Fig. 2). We found that the mutant exhibited only one tenth of the SOD activity compared to the parental strain, corroborating the specificity of the mutant. However, the assay also revealed that the SOD activity of the complemented mutant was about 10 times that of the parental strain confirming the Western blot results and arguing for a copy-number effect.

In line with a previous report [36], we further detected SOD2343 protein in the culture supernatant of the parental strains and the complemented mutants but not of the mutants (Figs. S2 and S3). In accordance with the results obtained with whole cell lysates, the amount of SOD2343 protein detected in the culture supernatant of complemented mutants was considerably above the level found in the supernatant of parental strains (Fig. S3).

### Effect of *sod2343* inactivation on surface-associated motility and growth

**Figure 3:**
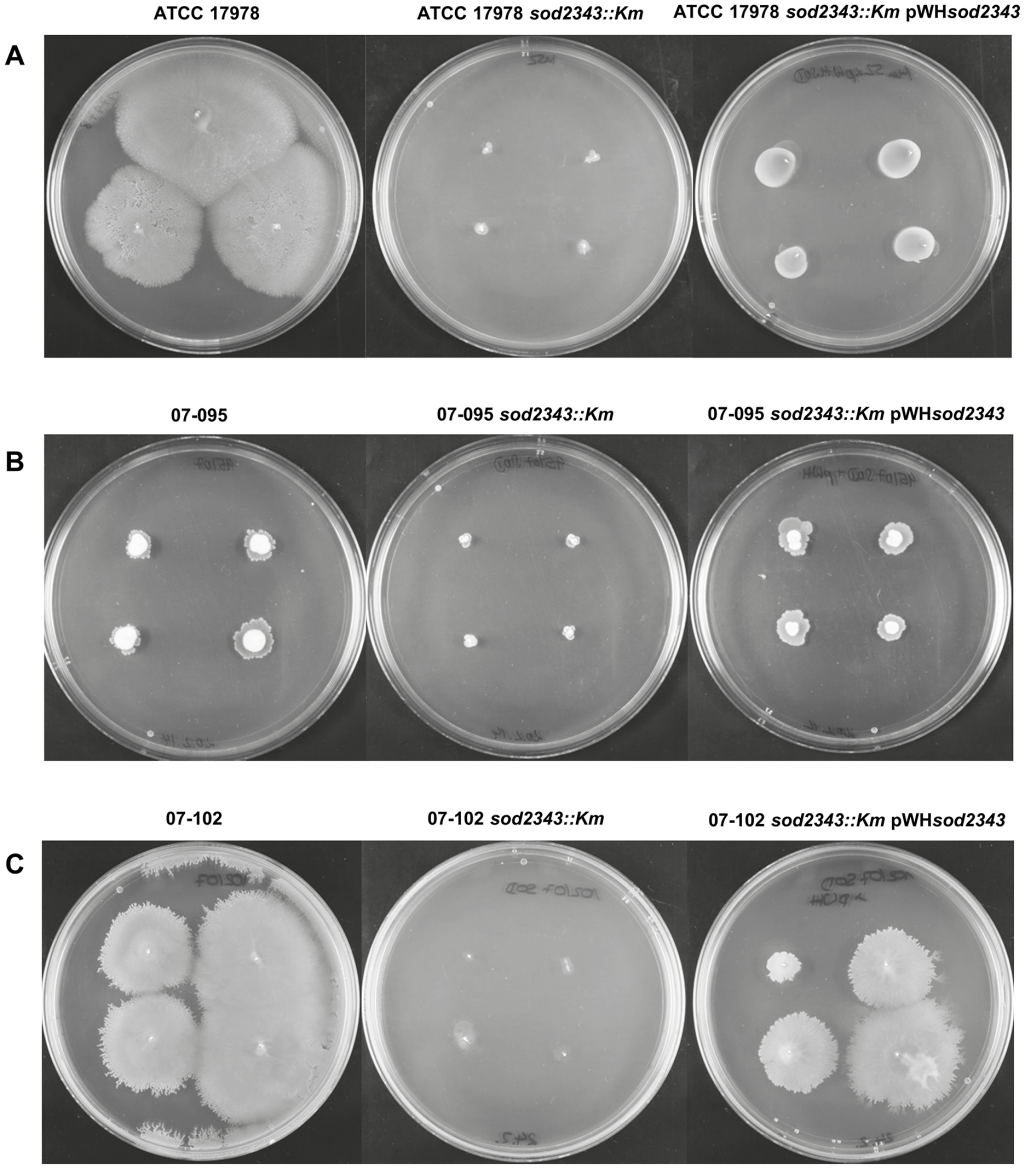
Motility assays on semi solid agarose plates (0.5%) reveal a motility defect of the *sod2343* mutants. (A) ATCC 17978 and ATCC 17978 *sod2343::Km*. (B) 07–095 and 07–095 *sod2343::Km* (C) 07–102 and 07–102 *sod2343::Km*. The pictures shown are representative of three independent replicates.

**Figure 4:**
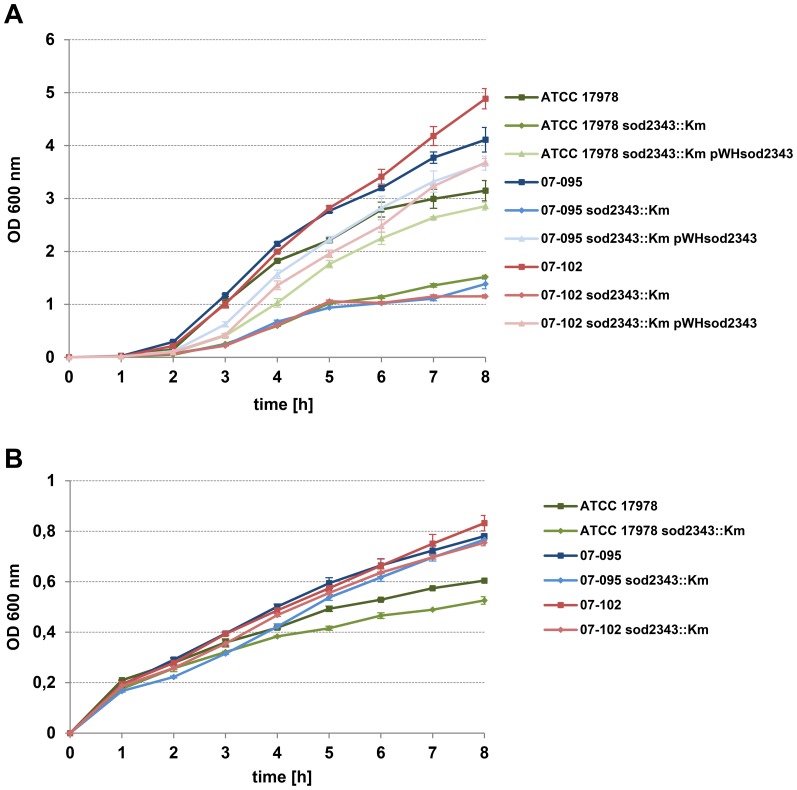
Growth defects of *sod2343* mutants depend on extent of aeration. Bacterial cultures as indicated were OD-adjusted from overnight cultures and grown at 37°C in LB medium under constant shaking for 8 hours either in baffled flasks (A) or in tubes (B) and the OD (600 nm) was determined. For each strain, data obtained from three independent cultures were averaged; error bars represent plus/minus one standard deviation. Growth of all *sod2343* mutants was significantly delayed compared to their parentals when grown in baffled flasks (A) (p<0.005, Student's *t* test,two-tailed, unpaired, for the last three time points). When grown in tubes (B), only the difference between ATCC 17978 and ATCC 17978 *sod2343* was significant (p<0.05).

Next, surface-associated motility of the *sod2343::Km* mutants was characterised in comparison to the parental strains ATCC 17978, 07–095 and 07–102 which are known to exhibit differing motility phenotypes on semi-solid media [38] (Fig. 3). In all three strains inactivation of *sod2343* completely abolished motility (Fig. 3). Interestingly, complementation of the mutants with pWH*sod2343* restored motility of 07–095 *sod2343::Km* (Fig. 3 B), and almost restored motility of 07–102 *sod2343::Km* (Fig. 3 C), whereas complementation of ATCC 17978 *sod2343::Km* resulted in only partial restoration accompanied by a change of the colony morphology (Fig. 3A). Since all mutants exhibited considerably reduced growth rates compared to their parental strains we prolonged incubation times of motility assays performed with the mutant strains. However, prolongation of incubation could not compensate the motility defects of the mutants (data not shown). We subsequently examined the growth behaviour of these strains in more detail. When cultured under intensive aeration in baffled flasks, all three *sod2343::Km* mutants exhibited a significant growth delay compared to their respective parental (Fig. 4A). Complementation of *sod2343::Km* mutants with pWH*sod2343* partially but not fully restored this growth inhibition in all three strains (Fig. 4A). By contrast, when grown under conditions of limited aeration in test tubes, growth defects of *sod2343::Km* mutants were less pronounced or even undetectable (Fig. 4B).

In a nutshell, all *sod2343::Km* mutants displayed significant motility defects when compared to their parental strains. Growth defects associated with these mutants depend on the extent of aeration and can thus be attributed to a reduced potential to detoxify ROS confirming the specificity of the mutations.

### Sensitivity of *sod2343* mutants to reactive oxygen species

Hydrogen peroxide and paraquat act as inducing reagents for reactive oxygen species (ROS). To elucidate the role of SOD2343 in protection against ROS, disc diffusion assays were conducted. Under all conditions tested *sod2343::Km* mutant strains were significantly more susceptible to H_2_O_2_ and to paraquat compared to their respective parental strains (p<0.005 for the highest concentration of H_2_O_2,_ and p<0.0005 for the paraquat concentration tested; Table 1 and Figs. S4 and S5). Interestingly, while complemented mutants showed an intermediate phenotype with regard to H_2_O_2_ exposition compared to mutants and parental strains (Table 1 and Fig. S4), complementation was more effective in protecting against paraquat leading to parental-like phenotypes in all strains (Table 1 and Fig. S5). Taken together, it can be concluded that, in line with its putative SOD activity, SOD2343 of *A. baumannii* significantly contributes to protection against ROS.

**Table 1:** Increased sensitivity of the *sod2434* mutants to H_2_O_2_ and paraquat exposure.

Strain	H_2_O_2_ [mm]	Paraquat [mm]
ATCC 17978	3	3.7
ATCC 17978 *sod2343::Km*	**6.7** *	**14.7** **
ATCC 17978 *sod2343::Km* pWH*sod2343*	5.3	2.5
07–095	1.7	0.3
07–095 *sod2343::Km*	**4.3** *	**7.2** *
07–095 *sod2343::Km* pWH*sod2343*	3.2	0.3
07–102	3.3	3.5
07–102 *sod2343::Km*	**4.7**	**12.8** **
07–102 *sod2343::Km* pWH*sod2343*	4.0	2.0

Average dimension of inhibition zone determined as the distance between paper disc and the front line of growth [mm], determined from three independent experiments. 5 µl of 10% H_2_O_2_ and 5 µl of 5 mg/ml paraquat, respectively, was deposited on a paper disc with a diameter of 5 mm; values given in **bold** indicate a significant difference between a given mutant and its corresponding parental strain (p<0.005; Student's t-test, two-tailed, unpaired;

* indicates p<0.0005;

** indicates p<0.00001).

### Effect of *sod2343* inactivation on susceptibility to antibiotics

Our understanding on how antibiotics kill bacteria is unfirm. While textbook knowledge claims independent mechanisms of killing of the different classes of bactericidal antibiotics, recently it was suggested that antibiotic action merges into a common pathway involving ROS in killing under aerobic conditions [30], [41]. Very recently, however, ROS-dependent killing has been refuted as a global mechanism of bactericidal activity [33], [34]. Here, we intended to elucidate a potential interrelationship between superoxide dismutase activity and susceptibility to antibiotics. Etest strips were used to determine and compare minimal inhibitory concentrations (MIC) of different antibiotics in relation to *sod2343* inactivation. Polymyxins such as colistin, an antibiotic of last resort for treatment of *A. baumannii* infections, have recently been reported to mediate killing in a ROS-dependent manner [32]. Here, we found that inactivation of *sod2343* resulted in a slight but reproducible (p<0.05) increase in sensitivity to colistin in all strains tested (Table 2 and Fig. S6). The mutants' increase in sensitivity was fully restored by complementation with pWH*sod2343* in all strains (Table 2 and Fig. S6). Exposure to gentamicin, by contrast, resulted in strain-dependent differences (Table 2 and Fig. S7). While sensitivity of ATCC 17978 *sod2343::Km* to gentamicin was increased compared to the parental strain (p<0.05) (Table 2 and Fig. S7A), sensitivity of 07–095 *sod2343::Km* was drastically reduced compared to parental 07–095 (p<0.05) (Table 2 and Fig. S7B). Similarly, sensitivity of 07–102 *sod2343::Km* to gentamicin was reduced compared to 07–102 (p<0.05) (Table 2 and Fig. S7C). Interestingly, the changes in sensitivity of the mutants towards gentamicin were fully restored by complementation with pWH*sod2343* in all strains irrespective of whether the mutants were more or less sensitive compared to their respective parentals (Table 2 and Fig. S7). Further, we observed strain-dependent responses to the antibiotics levofloxacin and imipenem (Table 2). ATCC 17978 *sod2343::Km* was significantly more susceptible to levofloxacin, an effect reversible by complementation, whereas no significant differences could be observed with strains 07–095 and 07–102 (Table 2). While mutants of ATCC 17978 and 07–095 proved more susceptible to imipenem, no significant difference was observed with 07–102. Next, we tested the susceptibility to tetracycline, a bacteriostatic antibiotic considered to act ROS-independent. Surprisingly, we reproducibly found the mutants of all strains to be more susceptible to tetracycline compared to their parental strains (p<0.005) (Table 2 and Fig. S8). The complemented mutants of all three strains were only partially restored with regard to tetracycline sensitivity. In conclusion, inactivation of *sod2343* interferes with susceptibility to antibiotics irrespective of their bactericidal or bacteriostatic activity.

**Table 2:** Minimal inhibitory concentrations determined by Etest.

Strain	Colistin MIC^3^	Gentamicin MIC^6^	Levofloxacin MIC^3^	Imipenem MIC^3^	Tetracycline MIC^6^
ATCC 17978	1.33	0.88	0.09	1.33	1.25
ATCC 17978 *sod2343::Km*	**0.75**	**0.38**	**0.05**	**0.25**	**0.31** *
ATCC 17978 *sod2343::Km* pWH*sod2343*	1.33	1	0.08	0.38	0.69
07–095	1.5	0.88	0.07	1.67	1.83
07–095 *sod2343::Km*	**0.83**	**2.92**	0.06	**0.46**	**0.44** *
07–095 *sod2343::Km* pWH*sod2343*	1.33	0.83	0.08	1.17	0.88
07–102	1.5	0.71	0.75	0.58	14.67
07–102 *sod2343::Km*	**0.92**	**1.92**	1.25	0.42	**6** *
07–102 *sod2343::Km* pWH*sod2343*	1.5	1	0.75	0.34	10.67

Average MIC values in [µg/ml] determined from three (MIC^3^) and six (MIC^6^) independent experiments, respectively; MIC value given in **bold** indicates that MIC value of mutant is significantly different from MIC value of the corresponding parental strain (p<0.05; Student's t-test, two-tailed, unpaired;

* indicates p<0.005).

### Role of *sod2343* in virulence of *A. baumannii* in the *Galleria mellonella* infection model

**Figure 5:**
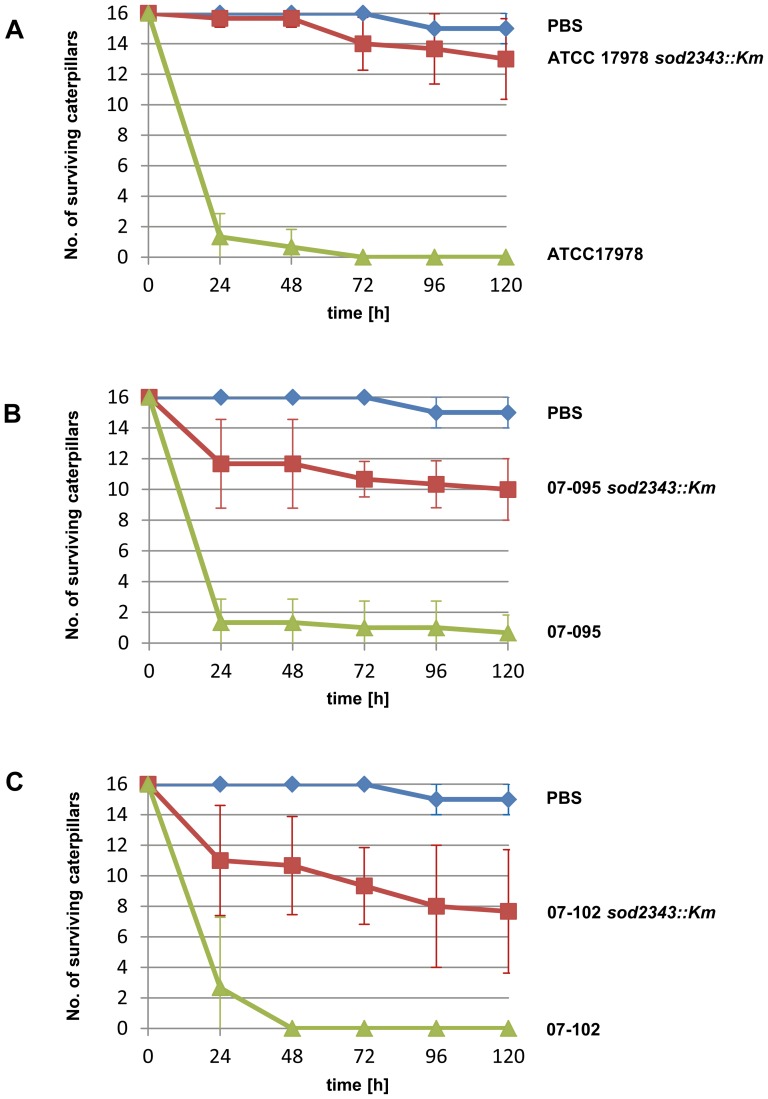
*A. baumannii sod2343* mutants are attenuated in the *Galleria mellonella* infection model. (A) Survival of *Galleria* caterpillars injected with 3×10^5^ CFU of ATCC 17978 *sod2343::Km* (red), ATCC 17978 (green) and PBS (blue). (B) Infection with 1.5×10^6^ CFU of 07–095 and its *sod2343::Km* derivative. (C) Infection with 3×10^5^ CFU of 07–102 and its *sod2343::Km* mutant. Results represent means and standard deviations of at least three independent experiments of 16 larvae per group.

**Figure 6:**
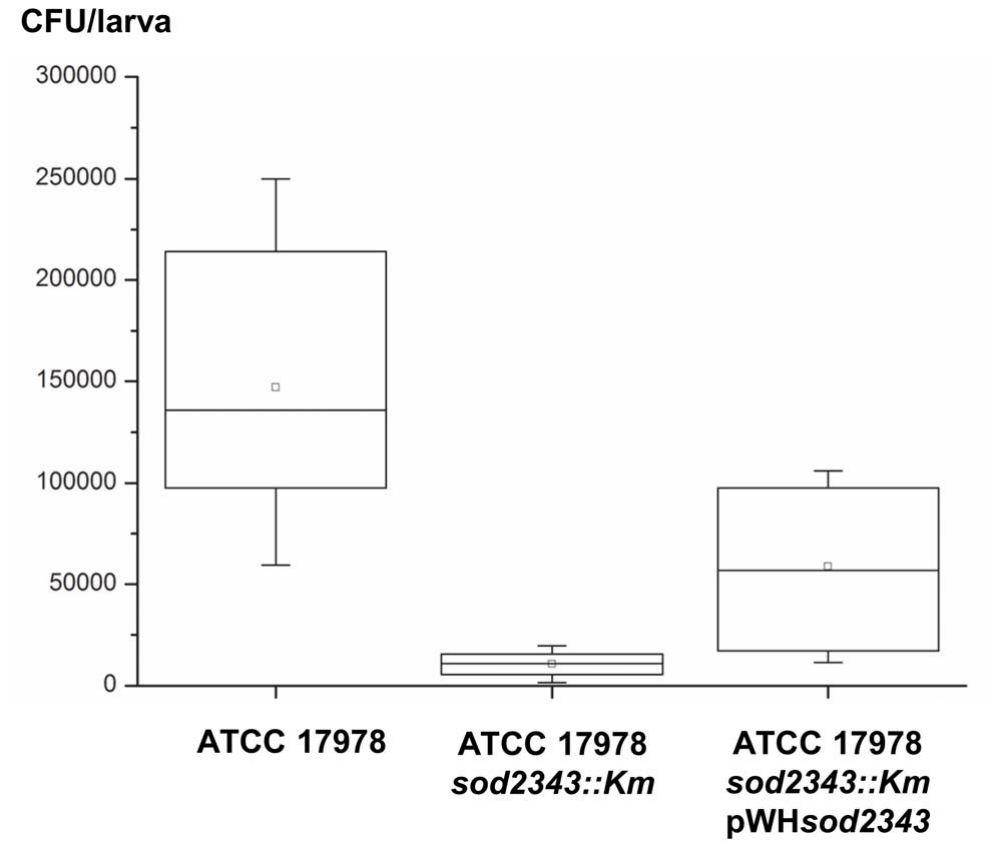
Recovery of *A. baumannii sod2343* mutant from *Galleria mellonella* larvae is minor compared to parental strain. Groups of 10 larvae were injected with 1.5×10^6^ CFU of ATCC 17978, ATCC 17978 *sod2343::Km*, and ATCC 17978 *sod2343::Km* pWH*sod2343*, respectively. Directly after injection of the 10 larvae within a group, the larvae were frozen at −80°C, homogenized and serial dilutions were plated on CHROMagar Acinetobacter to determine CFUs. Boxes indicate the 25–75% region, the median is indicated by a line in the box, the average is indicated by a small square, whiskers indicate maximum and minimum values, respectively.

The role of *sod2343* of *A. baumannii* in virulence was examined by making use of the *Galleria mellonella* caterpillar infection model (Fig. 5). The *sod2343::Km* mutants of strains ATCC 17978, 07–095 and 07–102 were all significantly attenuated compared to their respective parental strain (Fig. 5). The results suggest an important role of *sod2343* in virulence of *A. baumannii* isolates in *G. mellonella* infection. However, these data can be corrupted even by slightly different growth rates of parental and mutant strains. We therefore considered infection experiments of reduced duration such as gentamicin protection assays following infection of A549 human alveolar epithelial cells [42]. However, in our hands these infection experiments required several hours of infection to yield reliable recovery rates with our parental strains (data not shown). We subsequently examined the potential of using the *Galleria* model for short-time infection experiments and studied the recovery of bacteria shortly after their injection into the *Galleria* hemocoel. The bacteria were recovered by plating on CHROMagar Acinetobacter plates to suppress intrinsic microbiota of the larvae. We found that the average recovery of ATCC 17978 after 10 minutes was more than ten times that of the ATCC 17978 *sod2343::Km* mutant (p<0.0001) (Fig. 6). Interestingly, the complemented mutant (ATCC 17978 *sod2343::Km* pWH*sod2343*) exhibited an intermediate phenotype with recovery rates ranging between that observed for mutant and parental strain. Plasmid loss was excluded as an explanation for this intermediate phenotype as ten representative colonies recovered from CHROMagar Acinetobacter all harboured the plasmid without detectable rearrangements. Collectively, our data suggest that attenuation of *sod2343* mutants is primarily due to a lack of ROS detoxifying activity rather than due to reduced growth of the mutants.

## Discussion

Here, we have inactivated A1S_2343 of *A. baumannii* ATCC 17978 and the homologues of additional isolates to investigate the role of the putatively encoded FeSOD (SodB) in motility, resistance to ROS and antibiotics and its role in virulence. We proved by immuno-blotting that a protein of the expected size was expressed in the parental strains and complemented mutants but not in the mutants. In line with a potential contribution of SOD2343 to protection from ROS, we found that the *sod2343::Km* mutants were more susceptible to H_2_O_2_ and paraquat compared to their parental strains. On a first view, the significant impact of *sod2343* inactivation on motility seems to be attributable to the diminished potential to detoxify ROS when surface-exposed. However, we cannot rule out at present that sensing of the oxygen tension and/or ROS is transduced into signals controlling motility. At least, there is evidence of an interrelationship between type IV pilus-dependent motility and superoxide dismutase activity in phototaxis of cyanobacteria [43]. Moreover, in *Salmonella* a linkage between superoxide dismutase activity and flagella-driven motility was reported [44].

The outcome of our trans-complementation experiments was heterogeneous. While a number of *sod2343::Km*-associated phenotypes were fully restored by complementation, notably sensitivity to paraquat, colistin and gentamicin, other phenotypes were only partially restored in all mutants such as growth defects or sensitivity to H_2_O_2_ and tetracycline. Moreover, restoration was strain-dependent in some cases such as the motility defect of ATCC 17978 *sod2343::Km* that could not be restored while that of 07–095 and 07–102 mutants was remediable. We noted that the level of SOD expression in the complemented mutants significantly exceeded that of the parental strains. Given the complexity of redox homeostasis and associated ROS homeostasis [15], [45], it is not unexpected that imbalanced overexpression of SOD can lead to superimposed detrimental effects. It has been described, for example, that overproduction of an FeSOD in *E. coli* was associated with increased sensitivity to hyperoxic conditions and to paraquat but, interestingly, not to H_2_O_2_ [46]. Collectively, we attribute the incomplete complementation of specific strains under certain conditions to strain-specific differences in the redox and ROS homeostasis interfering with SOD overproduction. These findings call for the development of less disturbing complementation methods such as the use of low-copy number vectors and cis-complementation approaches that we intend to develop in the future.

Our infection experiments applying the *G. mellonella* model revealed a significant attenuation of all *sod2343* mutants. However, reduced killing of *Galleria* larvae by the *sod2343* mutants could be due to retarded proliferation within the host alone. On the other hand, recovery of mutant bacteria was drastically reduced immediately after infection suggesting a rapid host response to which mutants were more susceptible. As it is known that killing of bacteria by insect hemocytes involves the production of superoxide [47], our data suggest an important role of SOD2343 in protecting *A. baumannii* against ROS during infection. Superoxide dismutases (*sodA*, *sodB*, *sodC*) have been implicated in virulence in a number of pathogens characterized in various infection models (reviewed in [15]). Recently, *sodA* of *Enterococcus faecium* and *sodC* of *Yersinia pseudotuberculosis* were shown to contribute to resistance against host immune responses in *G. mellonella* [48], [49] underscoring the relevance of ROS defense in this model. However, the role of ROS in immune defense against *A. baumannii* in mammalian hosts is not fully understood. NADPH oxidase-deficient mice deficient in producing ROS have been shown to be more susceptible to infection with *A. baumannii* [28]. Strikingly, however, Qiu et al. found no indication of ROS-mediated killing of *A. baumannii* by macrophages *in vitro* [29]. It is interesting to note, that in *A. baumannii* expression of *sod2343* is stimulated by ethanol [37], an inducer which has been shown to induce several virulence-associated traits in this species [35]. This finding suggests that control of *sod2343* expression is integrated into a virulence-related regulon.

There is an ongoing debate on the contribution of ROS to the bactericidal activity of antibiotics under aerobic conditions irrespective of the antibiotics' primary target sites [30], [31], [33], [34]. Actually, several previous studies suggest an interrelationship between superoxide dismutase activity and the level of susceptibility to different classes of antibiotics although the literature is contradictory. An *E. coli sodA sodB* double mutant was reported to be less susceptible to bleomycin [41] and to kanamycin, ampicillin and norfloxacin [50]. Similar results were described for *E. coli* mutants by Feld et al. [51]. By contrast, Ezraty et al. could not observe significant differences between an *E. coli sodA sodB* double mutant and its parental strain regarding susceptibility to gentamicin and only marginal differences at intermediate ampicillin concentrations [52]. Interestingly, inactivation of the sole superoxide dismutase gene present in *Listeria monocytogenes* was found to have no impact on susceptibility to antibiotics [51]. The latter observation was explained by the incomplete TCA cycle found in *L. monocytogenes*, in line with the notion that ROS-dependent killing is coupled to TCA cycle activity [30]. In contrast to Wang et al. who reported no effect of either *sodA* or *sodB* inactivation on norfloxacin lethality [50], Dwyer et al. reported that a *sodB* mutant of *E. coli* was considerably more susceptible to norfloxacin compared to its parental strain [30], [31]. In *Campylobacter jejuni*, a *katA* mutant proved more sensitive to rifampicin and cefotaxime while the *sodB* mutant showed no effects, but a *sodB katA* double mutant was even more susceptible than the *katA* mutant [53]. Collectively, there is evidence of an interrelationship between ROS detoxifying activity and sensitivity to antibiotics. However, inactivation of related genes - if at all phenotypically relevant - can result in either increased or decreased sensitivity to antibiotics which, in our view, points to a very complex ROS homeostasis. Our data on the influence of *sod2343* inactivation on susceptibility to antibiotics are ambiguous, too. All *sod2343* mutants were more susceptible to colistin in line with the recently presented evidence that rapid killing of *A. baumannii* by polymyxins was associated with ROS [32]. Contrarily, susceptibility to gentamicin was either increased or decreased depending on the strain. Even more striking, all *sod2343* mutants exhibited increased susceptibility to tetracycline, an antibiotic considered bacteriostatic and therefore not supposed to act in a ROS-dependent manner. Interestingly, a study by Fajardo et al. [54] screening a transposon-generated library of *Pseudomonas aeruginosa* mutants for differential effects on resistance to various antibiotics identified amongst many others a *sodA* mutant that exhibited increased sensitivity to ciprofloxacin, tetracycline and ceftazidime while was more resistant to imipenem compared to the parental strain. We thus conclude that the classification of antibiotics as either “bactericidal” or “bacteriostatic” is not appropriate in the context of differentiation of the influences that SODs do have on resistance development and so may be conceptually misleading in interpreting the relation of antibiotic activity to ROS. Collectively, there is a clear phenomenological interrelationship between genes involved in ROS detoxification and susceptibility to antibiotics in *A. baumannii* as well as in other bacteria [30]–[32], [41], [50], [51], [53], [54]. On the other hand, the failure to correlate generation of ROS and antibiotic treatment reported very recently [33], [34], [52] calls for a very critical check-up of the notion of ROS-dependence of the bactericidal activity of antibiotics. ROS homeostasis involves more than 100 potentially ROS-producing enzymes in *E. coli* [45] making this a challenging task.

Regarding the two designated SODs of *A. baumannii*, biochemical studies should now prove whether the protein encoded by A1S_2343 is really an SOD of the Fe-type (SodB). Further, a mutant of the second *sod* gene in *A. baumannii* encoding a putative Cu-ZnSOD (*sodC*) should be generated and characterised likewise. We found here that only marginal residual SOD activity remained with the *sod2343* mutant suggesting a minor role of *sodC* or an induction under specific environmental conditions. In favour of the latter hypothesis, a recent report suggests a role of *sodC* down-regulation in contributing to colistin resistance in *A. baumannii* [55].

It is interesting to note that both SODs of *A. baumannii* have been detected in the culture supernatant recently [36]. Here, we have confirmed the presence of SOD2343 in the culture supernatant raising the question about the biological relevance of this finding. Recently, extracellular surface-exposed SOD has been demonstrated to contribute to survival of mycobacteria in macrophages and neutrophils [56]. We have thus tested whether the SOD2343 found in the culture supernatant was enzymatically active. However, in preliminary experiments we failed to demonstrate significant SOD activity in the supernatant. Future work should clarify whether SODs of *A. baumannii* occur in a surface-exposed form that is suitable to prevent oxidative damage.

## Supporting Information

Figure S1 PCR confirmation of *sod2343* inactivation.
(PDF)

Figure S2 Secretion of SOD2343 into the culture supernatant.
(PDF)

Figure S3 Secretion of SOD2343 after complementation of mutants.
(PDF)

Figure S4 Increased sensitivity of the *sod2343* mutants to H_2_O_2_ exposure.
(PDF)

Figure S5 Increased sensitivity of the *sod2343* mutants to paraquat exposure.
(PDF)

Figure S6 Increased sensitivity of the *sod2343* mutants to colistin.
(PDF)

Figure S7 Differential effects of *sod2343* inactivation on sensitivity to gentamicin.
(PDF)

Figure S8 Increased sensitivity of the *sod2343* mutants to tetracycline.
(PDF)
