# Supplementary material for: Impact of Acinetobacter baumannii Superoxide Dismutase on Motility, Virulence, Oxidative Stress Resistance and Susceptibility to Antibiotics
Source: PLoS One. 2014 Jul 7;9(7):e101033. doi: 10.1371/journal.pone.0101033 (PMC4085030; doi:10.1371/journal.pone.0101033)
Supplement: Figure S1 — PCR confirmation of sod2343 inactivation. (PDF) [file pone.0101033.s001.pdf]

Supplementary Fig. S1
Heindorf et al.

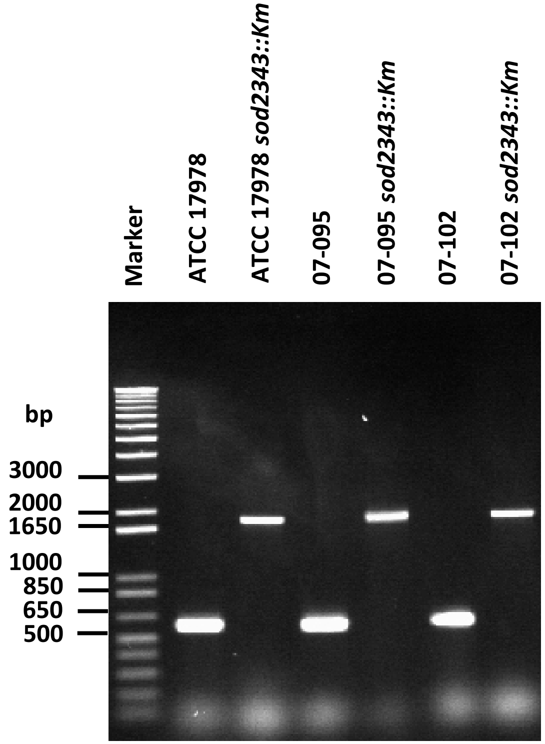

PCR confirmation of *sod2343* inactivation. *A. baumannii sod2343::Km* was generated by transposon mutagenesis (see Materials & Methods); the mutant's DNA was used for transformation of naturally competent isolates 07-095 and 07-102 of *A. baumannii* as described in Materials & Methods. Allelic exchange of *sod2343* with *sod2343::Km* was confirmed by PCR as described in Materials & Methods. The expected PCR product size derived from mutants is approximately 1800 bp and from parental strains is approximately 600 bp.
