# Supplementary material for: Impact of Acinetobacter baumannii Superoxide Dismutase on Motility, Virulence, Oxidative Stress Resistance and Susceptibility to Antibiotics
Source: PLoS One. 2014 Jul 7;9(7):e101033. doi: 10.1371/journal.pone.0101033 (PMC4085030; doi:10.1371/journal.pone.0101033)
Supplement: Figure S2 — Secretion of SOD2343 into the culture supernatant. (PDF) [file pone.0101033.s002.pdf]

Supplementary Fig. S2
Heindorf et al.

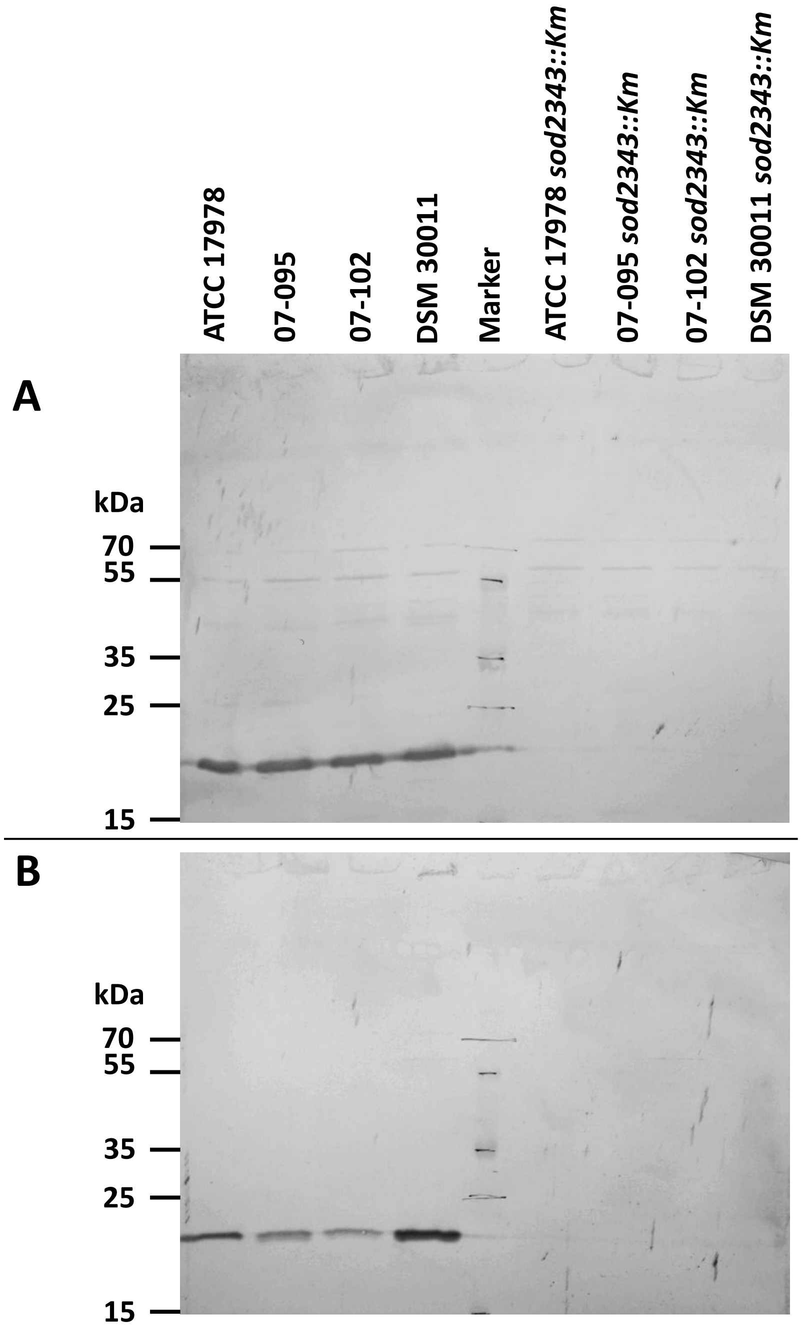

Secretion of SOD2343 into the culture supernatant. Bacterial cultures as indicated were diluted 1:50 into 3 ml of LB medium from overnight cultures and incubated at 37°C under constant shaking (150 rpm) for another 4 h. Cultures were adjusted to 1 $OD_{600\ nm}$, 0.5 ml of each was centrifuged and the pellet resuspended in 50 µl of SDS-PAGE loading buffer. 10 µl of each sample was loaded on an SDS-PAGE that was subsequently electro-blotted. A polyclonal antiserum raised against GST-SOD2343 fusion protein was diluted 1:5000 for detection (A). From the same OD-adjusted cultures 1.8 ml of supernatant was collected by centrifugation and precipitated with 10% TCA. The precipitated proteins were washed twice in ice-cold acetone, air-dried, resuspended in 30 µl of SDS-PAGE loading buffer and 10 µl of each sample was subjected to SDS-PAGE, electro-blotting and immunodetection as above. SOD2343 was detected in the culture supernatant of all parental strains but not of the *sod2343::Km* mutants (B).
