# Supplementary material for: Impact of Acinetobacter baumannii Superoxide Dismutase on Motility, Virulence, Oxidative Stress Resistance and Susceptibility to Antibiotics
Source: PLoS One. 2014 Jul 7;9(7):e101033. doi: 10.1371/journal.pone.0101033 (PMC4085030; doi:10.1371/journal.pone.0101033)
Supplement: Figure S3 — Secretion of SOD2343 after complementation of mutants. (PDF) [file pone.0101033.s003.pdf]

**Supplementary Fig. S3**
**Heindorf et al.**

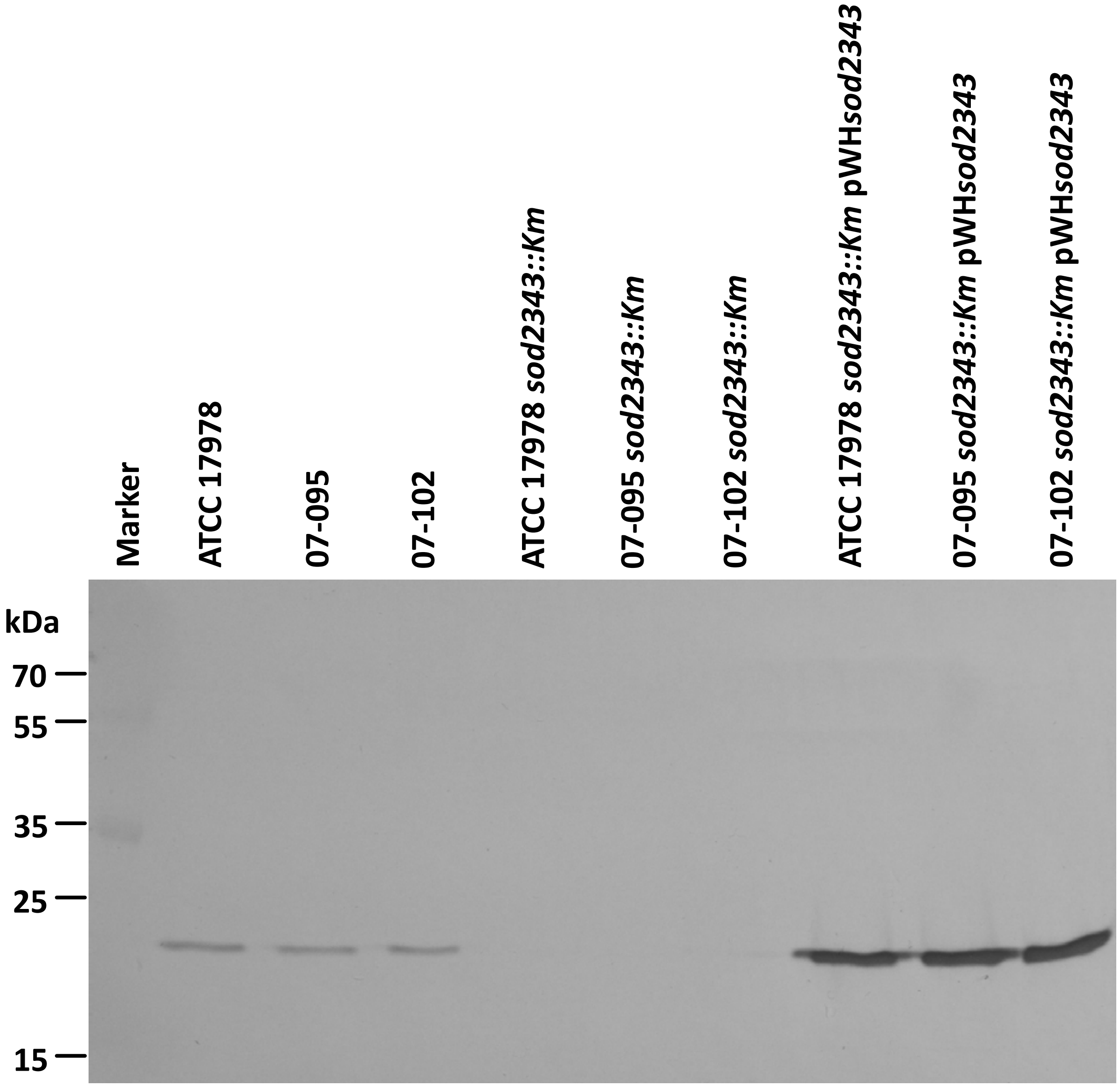


**Secretion of SOD2343 after complementation of mutants.** Overnight cultures as indicated were incubated at 37°C for 16 h under constant shaking (150 rpm) in LB broth. Cultures were adjusted to 1 OD at 600 nm and 1.8 ml of each supernatant was collected by centrifugation and precipitated with 10% TCA. The precipitated proteins were washed twice in ice-cold acetone, air-dried, resuspended in 30 µl of SDS-PAGE loading buffer and 10 µl of each sample was subjected to SDS-PAGE, electro-blotting and immunodetection. A polyclonal antiserum raised against GST-SOD2343 fusion protein was diluted 1:5000 for detection. SOD2343 was detected in the supernatant of all parental strains as well as the *sod2343::Km* mutants complemented with pWH*sod2343* but not in the supernatant of the *sod2343::Km* mutants.
