# Supplementary material for: Impact of Acinetobacter baumannii Superoxide Dismutase on Motility, Virulence, Oxidative Stress Resistance and Susceptibility to Antibiotics
Source: PLoS One. 2014 Jul 7;9(7):e101033. doi: 10.1371/journal.pone.0101033 (PMC4085030; doi:10.1371/journal.pone.0101033)
Supplement: Figure S4 — Increased sensitivity of the sod2343 mutants to H2O2 exposure. (PDF) [file pone.0101033.s004.pdf]

Supplementary Fig. S4
Heindorf et al.

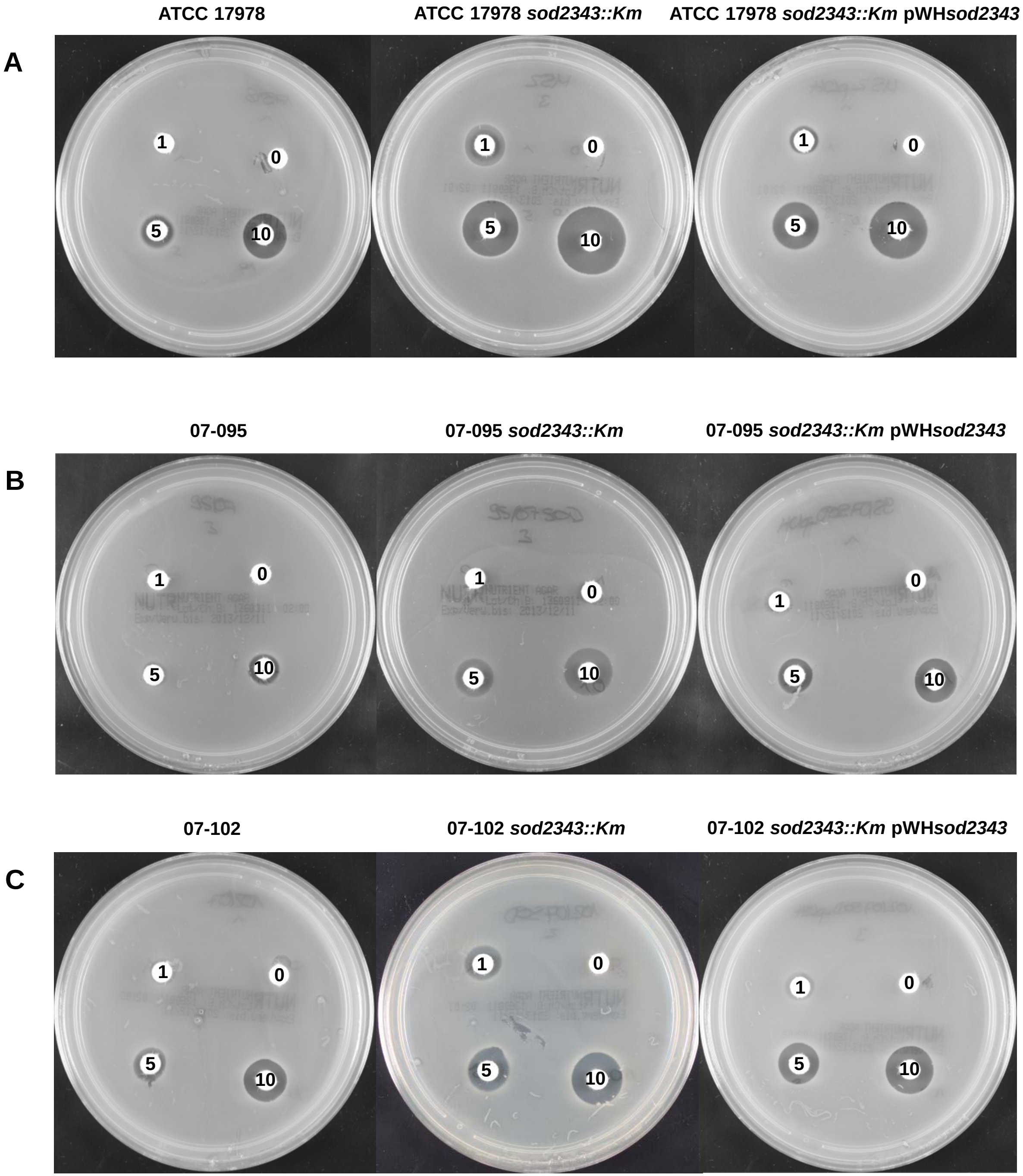

Increased sensitivity of the *sod2343* mutants to H_2O_2 exposure. Sensitivity to H_2O_2 exposure was determined with the disc diffusion method applying 5 µl of H_2O_2 solutions with concentrations of 0%, 1%, 5% and 10% H_2O_2 as indicated (see Materials & Methods). The pictures shown are representative of three independent replicates.
