# Supplementary material for: Impact of Acinetobacter baumannii Superoxide Dismutase on Motility, Virulence, Oxidative Stress Resistance and Susceptibility to Antibiotics
Source: PLoS One. 2014 Jul 7;9(7):e101033. doi: 10.1371/journal.pone.0101033 (PMC4085030; doi:10.1371/journal.pone.0101033)
Supplement: Figure S5 — Increased sensitivity of the sod2343 mutants to paraquat exposure. (PDF) [file pone.0101033.s005.pdf]

# Supplementary Fig. S5
## Heindorf et al.

**A**

ATCC 17978 | ATCC 17978 *sod2343::Km* | ATCC 17978 *sod2343::Km* pWH*sod2343*

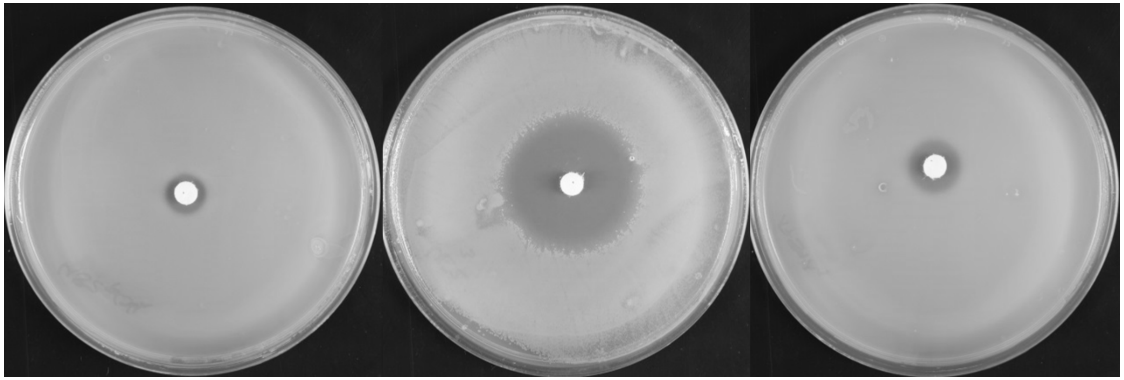

**B**

07-095 | 07-095 *sod2343::Km* | 07-095 *sod2343::Km* pWH*sod2343*

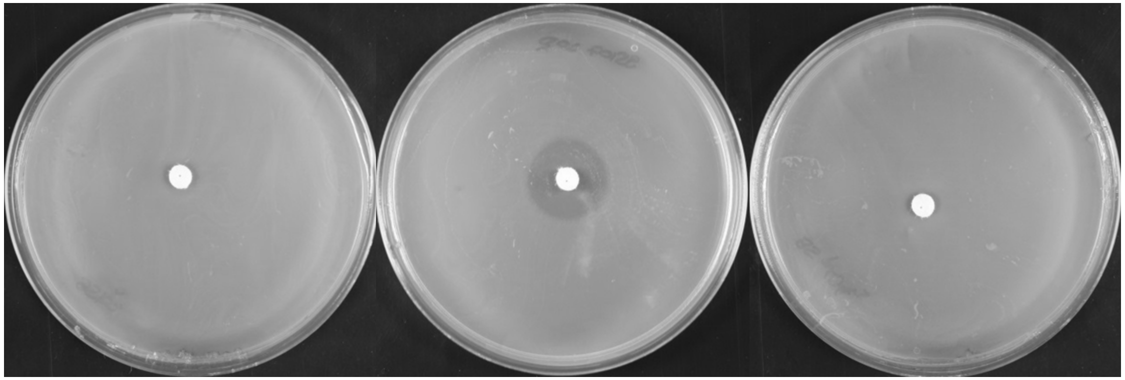

**C**

07-102 | 07-102 *sod2343::Km* | 07-102 *sod2343::Km* pWH*sod2343*

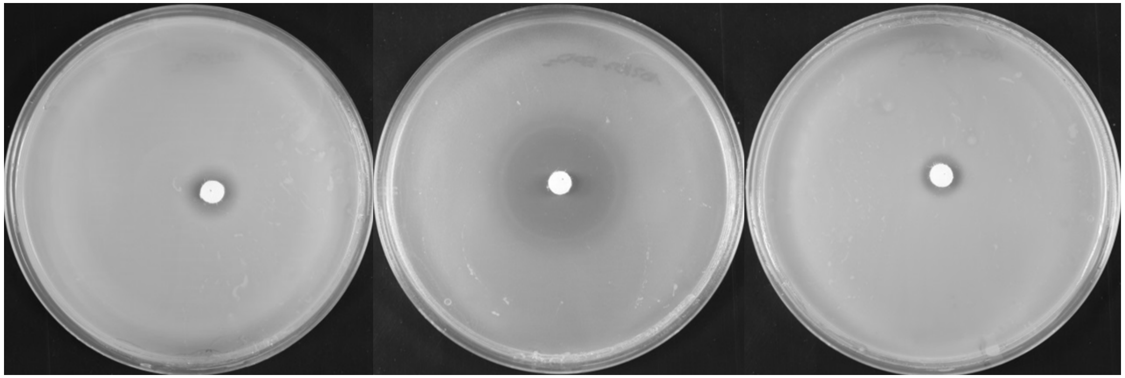

**Increased sensitivity of the *sod2343* mutants to paraquat exposure.** Sensitivity to paraquat exposure was determined with the disc diffusion method applying 5 µl of 5 mg/ml paraquat (see Materials & Methods). The pictures shown are representative of three independent replicates.
