# Supplementary material for: Impact of Acinetobacter baumannii Superoxide Dismutase on Motility, Virulence, Oxidative Stress Resistance and Susceptibility to Antibiotics
Source: PLoS One. 2014 Jul 7;9(7):e101033. doi: 10.1371/journal.pone.0101033 (PMC4085030; doi:10.1371/journal.pone.0101033)
Supplement: Figure S6 — Increased sensitivity of the sod2343 mutants to colistin. (PDF) [file pone.0101033.s006.pdf]

Supplementary Fig. S6
Heindorf et al.

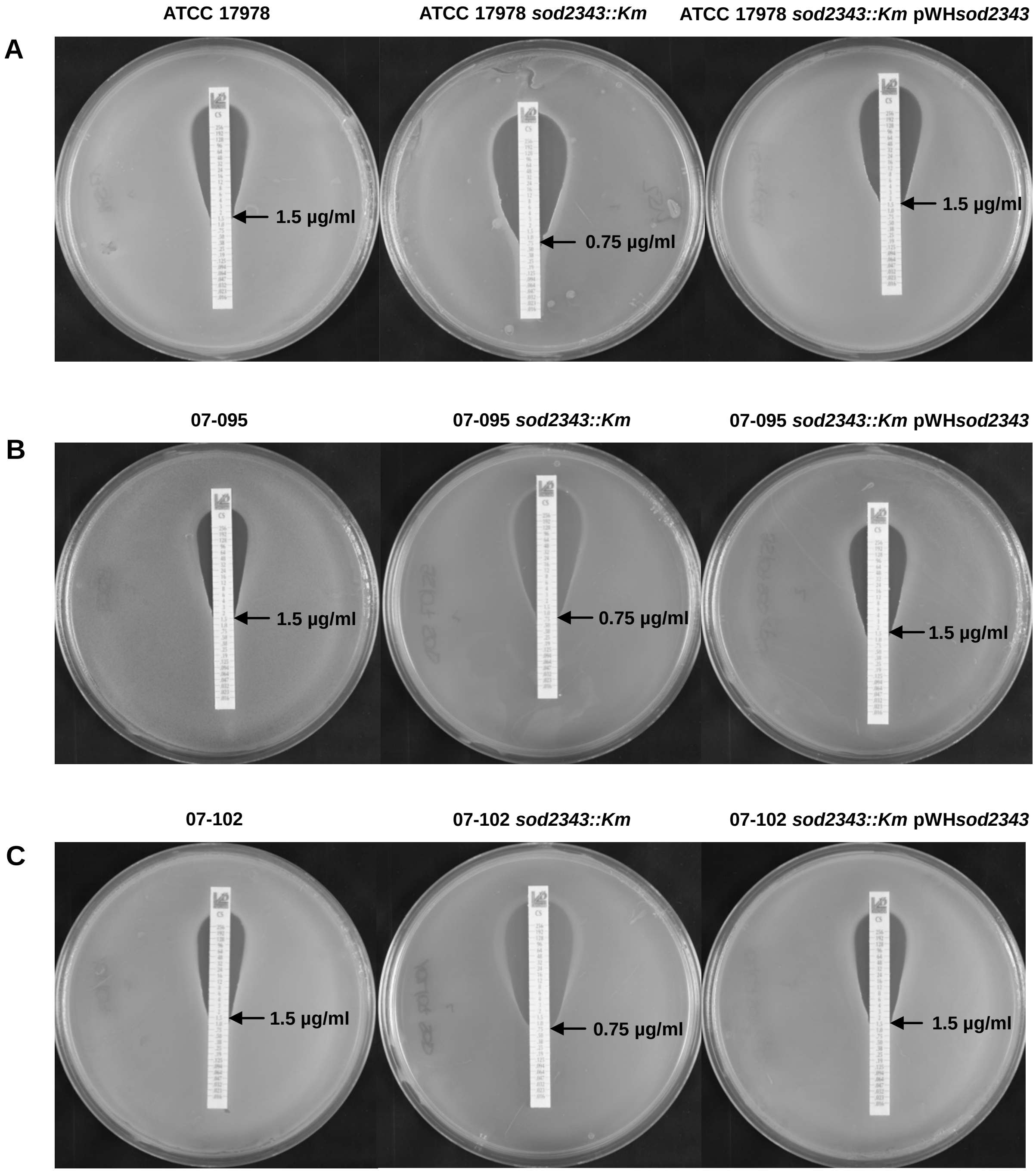

Increased sensitivity of the *sod2343* mutants to colistin. Etest strips were used to determine the minimal inhibitory concentration of colistin for *sod2343::Km* mutants, mutants complemented with pWH1266*sod2343* and parental strains as indicated (see Materials & Methods). The pictures shown are representative of three independent replicates (see also Table 2).
