# Supplementary material for: Impact of Acinetobacter baumannii Superoxide Dismutase on Motility, Virulence, Oxidative Stress Resistance and Susceptibility to Antibiotics
Source: PLoS One. 2014 Jul 7;9(7):e101033. doi: 10.1371/journal.pone.0101033 (PMC4085030; doi:10.1371/journal.pone.0101033)
Supplement: Figure S7 — Differential effects of sod2343 inactivation on sensitivity to gentamicin. (PDF) [file pone.0101033.s007.pdf]

Supplementary Fig. S7
Heindorf et al.

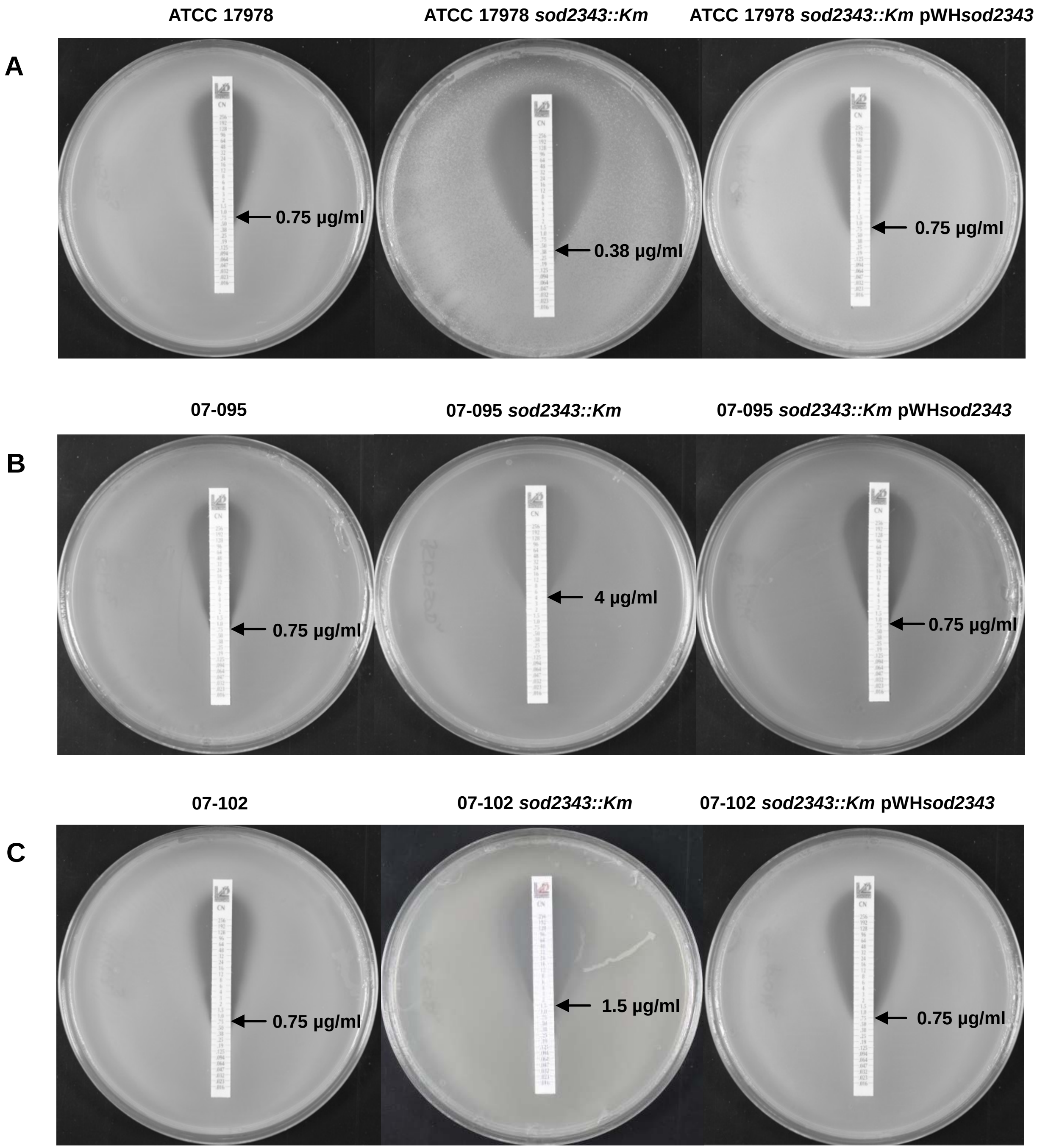

Differential effects of *sod2343* inactivation on sensitivity to gentamicin. Etest strips were used to determine the minimal inhibitory concentration of gentamicin for *sod2343::Km* mutants, mutants complemented with pWH1266*sod2343* and parental strains as indicated (see Materials & Methods). The pictures shown are representative of six independent replicates (see also Table 2).
